# Scedosporium-induced keratitis: insights from a case study

**DOI:** 10.1186/s12348-025-00533-2

**Published:** 2025-09-26

**Authors:** Liya Fathima, L. Annapurneswari, Pooja Rao, Teena Mendonca

**Affiliations:** 1 Department of Microbiology, Kasturba Medical College Mangalore, Manipal Academy of Higher Education, Manipal, India; 2 Department of Ophthalmology, Kasturba Medical College Mangalore, Manipal Academy of Higher Education, Manipal, India

**Keywords:** *Scedosporium apiospermum*, *Pseudallescheria Boydii*, Keratitis, Eye injury

## Abstract

**Introduction:**

*Pseudallescheria boydii* with its asexual form, *Scedosporium apiospermum*, is now recognized as an important emerging opportunistic pheoid-hypomycosis. Usually results in invasive infections in immunocompromised patients but rarely can infect the eyes. Herein, we report a challenging case of *Pseudallescheria boydii* keratitis in a lady with traumatic injury.

**Case Report:**

A 38-year-old female presented with alleged history of penetrating injury to right eye with stick by her spouse for which she was started on antibiotic eye drops from nearby local hospital and became symptomatically better. Later she had alleged history of ashes thrown to her right eye 2 weeks ago. She was referred here from a nearby hospital, with complaints of pain, itching, watering of eye and blurring of vision. On examination, Limbal -limbal ulcer with peripherally thinned out cornea and mucoid discharge was present. Corneal scrapings revealed *P boydii*. She was managed with antibiotics such as moxifloxacin, tobramycin and antifungals such as Natamycin, fluconazole. She did not improve symptomatically. She was referred to a higher center for keratoplasty and the patient was thus discharged for the same.

**Conclusion:**

*Pseudallescheria boydii* is a soil saprophytic, uncommon fungi entering body via respiratory or penetrating injury. It is often mistaken as Aspergillosis, This fungi shows resistance to amphotericin B, flucytosine and susceptible to triazoles. Surgical resection of lesion shows better outcome. The response of disseminated infection to combination therapy with interferon gamma and antifungal agents is encouraging.

##  Introduction

*Pseudallescheria boydii* with its asexual form, *Scedosporium apiospermum*, is now recognized as an important emerging opportunistic pheoid-hypomycosis. This filamentous fungi is found ubiquitous in soil, water, and sewage. It usually results in invasive infections in immunocompromised patients of various organs such as central nervous system, respiratory and cardiovascular systems. But rarely can infect the eyes [1].

Herein, we report a challenging case of *Pseudallescheria boydii* keratitis in a lady with traumatic eye injury.

### Case presentation

A 38-year-old female presented with alleged history of penetrating injury to right eye with a wooden stick by her spouse. She was diagnosed with Right eye pankeratitis for which she was prescribed Natamycin & Moxifloxacin eye drops and Tab Fluconazole 150 mg stat at a nearby local hospital and became symptomatically better. Later she had again alleged history of ashes thrown to her right eye 2 weeks back. She was referred here from the nearby hospital, with complaints of pain, itching, watering eye and blurring of vision.

**Fig. 1:**
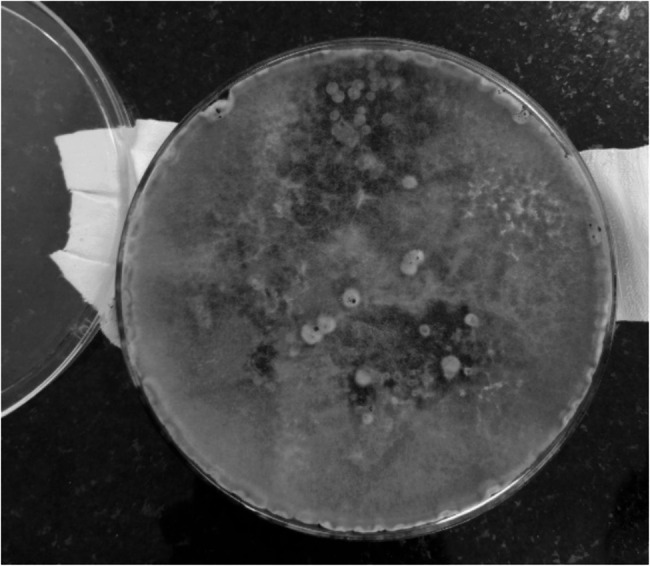
SDA showing macroscopic colony morphology of the mould

On Examination of right eye, A Limbal -limbal ulcer with peripherally thinned out cornea and mucoid discharge was noted. Hypopyon measuring 3–4 mm, epithelial defect 10 × 9 mm and stromal infiltration was present with no corneal sensation. Lens was clear with no opacities. Vision of right eye perceived light and presented with no accommodation correction with central corneal clarity. Outward deviation of right eye observed. Rest anterior chamber details and extraocular movements of both eyes were normal with patent sac. Left eye examination was within normal limits with vision 6/6. No history of known comorbidities. Corneal scrapings were sent. KOH mount showed slender septate hyphae with brown pigment. Sabouraud`s dextrose Agar (SDA) culture showed white cottony aerial mycelium turning white to grey. Reverse pigment was at first white later turned black in colour [2] (Fig. 1).

**Fig. 2:**
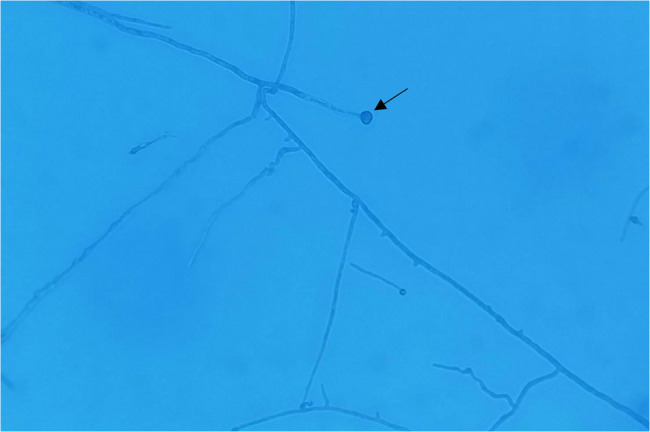
Microscopic morphology on LPCB mount showing lollipop appearance conidia

Lacto phenol Cotton Blue (LPCB) mount showed septate hyphae with conidiophores bearing oval or club shaped single conidia with “lollipop appearance”. (Fig. 2). Corneal scrapings thus revealed *P. boydii* after 1 week of incubation and was identified based on macroscopic, microscopic morphology and MALDI-ToF. The sexual state (Cleistothesia) was not seen.

Based on her clinical picture, she was managed with antibiotics such as Moxifloxacin eye drops, 10 times daily, Natamycin eye drops 4 times a day, Atropine 1% eye drops twice daily and antifungal Tab fluconazole 150 mg twice daily. She did not improve symptomatically. The antibiotics were continued for 3 days and she was referred to a higher center for therapeutic penetrating keratoplasty and the patient was thus discharged for the same.

##  Discussion

*Pseudallescheria boydii* is a soil saprophytic, uncommon fungi entering the body via respiratory or penetrating injury. It is often mistaken as Aspergillosis, this fungus shows resistance to amphotericin B, flucytosine and susceptible to triazoles hence early detection and appropriate therapeutic measures is the key for successful outcome. Natamycin is the first line treatment for fungal keratitis. Only a few studies have shown improved outcomes with Natamycin as monotherapy [3, 4]. In Ziqiang et al. study, one of the patients showed improvement with Natamycin with Amphotericin B unlike our case where in the prognosis was poor despite of timely initiation of Natamycin and Fluconazole probably due to deeper tissue involvement or drug resistance.

The presence of hypopyon indicates the posterior chamber involvement leading to failure of antifungal therapy and timely surgical intervention with therapeutic penetrating keratoplasty would determine the prognosis in such instances [5]. From most of the documented evidence the source of the infection was dust, vegetative matter or infection following laser in situ keratomileusis as seen in our study where ash dust formed the source [6]. Ali Tabatabaei et al. discussed that 2 rare causes of filamentous fungal keratitis in farmers with ages 33 and 56 yrs respectively along with a history of ocular trauma by plants were *P.boydii* and *Colletotrichum coccodes* [7]. Some of the emerging microorganisms causing infectious keratitis in the review of Pranita et al. were *Nocardia species*,* Chrysobacterium species*,* Kocuria species*,* Enterococcus species*,* Bartonella henslae*,* Achromobacter species and others* [8]. Christine et al., reviewed that Infectious keratitis represents significant health concern, ranking as the *5th* leading cause of blindness. Elderly people are more susceptible due to age related changes in immune response and corneal structure [9]. *Scedasporium* can cause a wide range of infections from sinusitis, keratitis, brain abscess and subcutaneous skin and soft tissue infections, in immunocompromised as well as immunocompetent hosts [7]. Tobias Lahmer et al. reported an unusual case of *P.boydii* pneumonia in a 69-year-old critically ill immunocompromised patient with *A.fumigatus and A.terrus*. It mainly highlighted the importance of superimposed fungal infections in critically ill and immunocompromised [1]. Bacteria and fungi which are commonly associated with endophthalmitis unlike our case study with pan keratitis following penetrating injury includes *Staphylococcus aureus*, *Bacillus*,* Streptococcus*, *Clostridium*,* Pseudomonas species*, *Microsporium species*,* Candida species*, *Aspergillus*,* Fusarium*,* Dematiaceous fungi and Paecilomyces* [10, 11]. The response of disseminated infection to combination therapy with interferon gamma & antifungal agents is encouraging [12]. Collagen cross linking is beneficial for fungal keratitis cases as a standalone therapy or adjunct to antifungal medications [13]. Limitations was molecular confirmation of the identified organism and antifungal susceptibility testing to give targeted therapy was not in scope of the present study.

## Conclusion

*Pseudallescheria boydii* is most important cause of fungal keratitis in immunocompetent people. Early suspicion and diagnosis of the fungal aetiology with appropriate medical/surgical treatment can have improved outcomes with preserving of vision and complications.

## Data Availability

No datasets were generated or analysed during the current study.
